# Comparative phylogenetic analysis of CBL reveals the gene family evolution and functional divergence in *Saccharum spontaneum*

**DOI:** 10.1186/s12870-021-03175-3

**Published:** 2021-08-23

**Authors:** Xiaomin Feng, Yongjun Wang, Nannan Zhang, Shuai Gao, Jiayun Wu, Rui Liu, Yonghong Huang, Jisen Zhang, Yongwen Qi

**Affiliations:** 1 Guangdong Sugarcane Genetic Improvement Engineering Center, Institute of Nanfan & Seed Industry, Guangdong Academy of Sciences, Room 1909, Biological Engineering Building, Jianghai Avenue, Haizhu District, Guangzhou, 510316 Guangdong Province China; 2 Center for Genomics and Biotechnology, Fujian Provincial Key Laboratory of Haixia Applied Plant Systems Biology, College of Agriculture, Fujian Agriculture and Forestry University, Fuzhou, 350002 China; 3 Guangxi Key Laboratory of Sugarcane Genetic Improvement, Nanning, 530007 China

**Keywords:** *CBL*, Evolution, *S. spontaneum*, Low K^+^ stress, Gene expression

## Abstract

**Background:**

The identification and functional analysis of genes that improve tolerance to low potassium stress in *S. spontaneum* is crucial for breeding sugarcane cultivars with efficient potassium utilization. Calcineurin B-like (CBL) protein is a calcium sensor that interacts with specific CBL-interacting protein kinases (CIPKs) upon plants’ exposure to various abiotic stresses.

**Results:**

In this study, nine *CBL* genes were identified from *S. spontaneum*. Phylogenetic analysis of 113 *CBLs* from 13 representative plants showed gene expansion and strong purifying selection in the *CBL* family. Analysis of *CBL* expression patterns revealed that *SsCBL01* was the most commonly expressed gene in various tissues at different developmental stages. Expression analysis of *SsCBLs* under low K^+^ stress indicated that potassium deficiency moderately altered the transcription of *SsCBLs*. Subcellular localization showed that *SsCBL01* is a plasma membrane protein and heterologous expression in yeast suggested that, while SsCBL01 alone could not absorb K^+^, it positively regulated K^+^ absorption mediated by the potassium transporter SsHAK1.

**Conclusions:**

This study provided insights into the evolution of the *CBL* gene family and preliminarily demonstrated that the plasma membrane protein SsCBL01 was involved in the response to low K^+^ stress in *S. spontaneum*.

**Supplementary Information:**

The online version contains supplementary material available at 10.1186/s12870-021-03175-3.

## Background

Sugarcane cultivars (*Saccharum* spp.) are mainly grown in tropical and subtropical regions of the world. Potassium leaching and soil acidification are common in these regions, thus decreasing the soil potassium content in sugarcane cultivation regions, particularly if the cultivation layer is low. A lack of soil potassium adversely affects the yield and quality of sugarcane [1]. Calcineurin B-like (CBL) protein, a plant calcium-binding protein initially identified in Arabidopsis, is a member of a group of small proteins that are strongly homologous with the regulatory B subunit of calcineurin in yeast [2]. When the cells of plant roots perceive reduced K^+^ concentrations in the external environment, Ca^2+^ signals are generated and relayed by CBLs to activate K^+^ channel proteins and potassium transporters, enhancing the uptake and utilization of K^+^ [3].

Subsequent studies have revealed that rice (*O. sativa* L.) and Arabidopsis (*A. thaliana*) possess 10 distinct CBL proteins [4, 5]. Using comparative genomic methods, different numbers of CBLs have been identified in maize (*Z. mays* L.), sorghum (*S. bicolor* L.), poplar (*Populus* L.), and cotton [6–9]. All CBLs are characterized by the presence of at least three conserved calcium-binding EF (elongation factor)-hand motifs. The EF-hand motif is defined by its helix–loop–helix secondary structure as well as in the ligands presented by the loop to bind the Ca^2+^ ion [10]. To relay Ca^2+^ signals, CBLs interact with target proteins, such as kinases, cytoskeletal-associated proteins, and metabolic enzymes to regulate gene expression [11, 12]. CBL-interacting protein kinases (CIPKs) are important target proteins of CBLs [3]. The CBL-CIPK complex has an indispensable role in plant response to abiotic stresses such as salinity, potassium starvation, low temperature, and drought [2, 13]. Besides, it also serves as an important signaling network regulating growth and development, uptake and transport of NO_3_^−^, NH_4_^+^, and iron, H^+^ homeostasis, and reactive oxygen species (ROS) signal transduction in plants [13, 14].

To date, studies on the CBL family have mainly focused on Arabidopsis, rice, and maize. In Arabidopsis, *AtCBL1/9* was first reported to positively regulate K^+^absorption by forming a CBL1/9-CIPK23 complex and activating the inward-rectifier K^+^channel AKT1 (Arabidopsis K^+^ transporter 1) by phosphorylation [15, 16]. The AtCBL1-AtCIPK23 complex also affects abscisic acid (ABA)-induced stomatal aperture and ROS signaling [17, 18]. The AtCBL2-AtCIPK11 complex has been recognized to have a negative role in activating the plasma membrane H^+^-ATPase (PMA) [19, 20]. AtCBL2/3 was found to be involved in protecting plants from high Mg^2+^ toxicity by regulating the sequestration of Mg^2+^in vacuole by forming a multivalent network with AtCIPK3/9/23/26 in Arabidopsis [21]. AtCBL2/3-CIPK12 complexes localized on the tonoplast participate in the controlling the germination of pollen grains and tube growth [22]. The AtCBL4-CIPK6 complex is a crucial regulator for the translocation of AtAKT2 from the endoplasmic reticulum to PM [23]. Both AtCBL4 and AtCBL10 are involved in the salt stress response through activation of AtCIPK24. *AtCBL4* is primarily expressed in the root tissues under high-salt conditions [24], while *AtCBL10* is expressed in leaves [25]. The AtCBL4-AtCIPK24 complex functions at the plasma membrane to drive the Na^+^/H^+^ exchanger SOS1 to extrude Na^+^ out of the cell to improve salt tolerance [24]. The AtCBL10-AtCIPK24 complex functions at the tonoplast and stimulates the Na^+^/H^+^ exchanger to sequestrate Na^+^ into the vacuole in the case of salt toxicity [25]. In rice, the homologous OsCBL4 protein has been found to interact with OsCIPK24 and participates in the SOS signaling pathway in response to salt stress [26]. The OsCBL1-OsCIPK23 complex also enhances OsAKT1-mediated K^+^ uptake in rice roots [27]. In maize, ZmCBL9 can interact with eight maize CIPKs, i.e., ZmCIPK8/9/15/23/24/31/32/39, to regulate abiotic stress including dehydration, salt, ABA, and low K^+^level [8].

Sugarcane is the chief sugar and biofuel feedstock crop, contributing 80 and 40% of the world’s sugar and ethanol, respectively [28]. Sugarcane cultivars are interspecific hybrids of *S. officinarum* (2*n =* 8 x = 80, x = 10) and the wild species *S. spontaneum* with many aneuploid forms (2*n =* 5 x ~ 16 x = 40 ~ 128; x = 8) and various cytotypes [29]. *S. officinarum* contributes to the high sugar content and *S. spontaneum* is added for incorporating hardiness, resistance against diseases, and ratooning capacity by backcrossing to *S. officinarum* to recover its high sugar content as well as high biomass [29], which is a major breakthrough in sugarcane breeding history. Consequently, the sugarcane cultivars are interspecific hybrids, polyploid, aneuploid with around 80% chromosomal content derived from *S. officinarum*, 10 ~ 15% from *S. spontaneum* and the remaining 5 ~ 10% from interspecific recombinants [30, 31]. Genes of the *CBL* family have been reported to be engaged in the regulation of the low potassium response in Arabidopsis, rice, and maize [4, 5, 8] but their roles and mechanisms of regulation in sugarcane remain unknown. This study revolving around the recently released *S. spontaneum* genome [32], comprehensively analyses the *CBL* family in *S. spontaneum* and other plants. The expression patterns of *SsCBLs* were monitored during development and in response to low K^+^ stress. *SsCBL01* was selected for further functional analysis. Taken together, this study performed a systematic analysis of the evolution of the *CBL* family and identified some robust *CBL* genes as candidates for responding to low K^+^ stress in sugarcane.

## Results

### Recognition of *CBL* genes in *S. spontaneum*

A total of nine distinct *SsCBLs* were identified from the AP85–441 tetraploid *S. spontaneum* genome [32] by excluding alleles. Eight *SbCBLs* were correspondingly identified from *S. bicolor*, the closest relative of sugarcane, based on comparative genomics (Table 1). For consistency, these *SsCBLs* were named according to the *CBL* nomenclature in *O. sativa* [4] and phylogenetic relationships. Each *SsCBL* has 1 to 4 alleles with a mean value of 3 in the *S. spontaneum* genome (Table S1). Among the nine *SsCBLs*, *SsCBL01* and *SsCBL03* were located on chromosome 1; *SsCBL05*, *SsCBL09,* and *SsCBL10* were located on chromosome 3; *SsCBL04* and *SsCBL06* were located on chromosome 7; *SsCBL02* was located on chromosome 2, and *SsCBL08* was located on chromosome 4. All *SsCBLs* have 8 exons except for *SsCBL09* and encode 211 to 294 amino acid residues. The predicted subcellular location of SsCBLs was the plasma membrane (PM), consistent with their role as calcium sensors to sense and decode extracellular signals. In addition, some SsCBLs may be also located on the tonoplast or in the cytoplasm. Sequence alignment of SsCBLs with their *S. bicolor* orthologs illustrated that the identities ranged from 78.54 to 100%, with an average of 95.15% (Table 1).

**Table 1:** Overview and comparison of *CBL* genes in *S. bicolor* and *S. spontaneum*

*S. bicolor*						*S. spontaneum*							Similarity^e^
Gene	Chr.^a^	Aa^b^	No. of exon	Mw^c^ (kDa)	P.L.^d^	Gene	Clade	Chr.^a^	Aa^b^	No. of exon	Mw^c^ (kDa)	P.L.^d^	
Sobic.001G294300	1	213	8	25.11	PM	*SsCBL01*	III	1	213	8	24.49	PM	100.00%
Sobic.008G152800	8	225	8	25.84	PM/Tono	*SsCBL02*	IV	2	225	8	25.84	PM/Tono	97.35%
NA	NA	NA	NA	NA	NA	*SsCBL03*	IV	1	225	8	25.77	PM/Tono	NA
Sobic.009G210300	9	212	8	24.05	PM	*SsCBL04*	I	7	211	8	24.11	PM/Cyto	78.54%
Sobic.003G208400	3	218	8	25.82	PM/Cyto	*SsCBL05*	I	3	220	8	25.66	PM/Cyto	96.98%
Sobic.008G046500	8	223	8	25.68	PM	*SsCBL06*	IV	7	223	8	25.68	PM	99.11%
Sobic.004G130600	4	213	8	24.39	PM/Cyto	*SsCBL08*	I	4	213	8	24.34	PM/Cyto	99.07%
Sobic.003G196400	3	319	9	35.87	PM	*SsCBL09*	II	3	294	9	33.11	PM	94.59%
Sobic.003G275000	3	283	8	32.29	PM/Tono	*SsCBL10*	II	3	288	8	32.4	PM/Tono	87.59%

*PM* plasma membrane, *Tono* tonoplast, *Cyto* cytoplasm.

^a^Chromosome carrying the target gene

^b^Number of amino acids of CBL protein sequence

^c^Prediction of Molecular weight (Mw) through ExPASy (https://web.expasy.org/compute_pi/)

^d^Subcellular location of the HAK proteins predicted with WoLF PSORT (https://www.genscript.com/wolf-psort.html)

^e^Similarity of protein sequence between *S. spontaneum* and sorghum determined by BLASTP

**Fig. 1:**
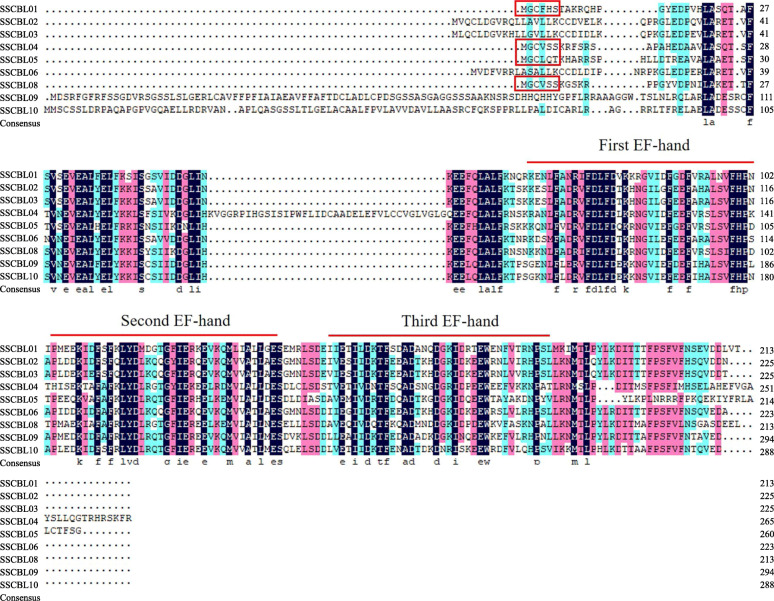
Amino acid sequence alignment of SsCBLs by DNAMAN software. The degree of similarity of the nine SsCBLs protein sequences is shown in distinct colors (50–74% cyan; 75–99%, cherry red; deep blue, 100%). EF-hand domains are indicated above the alignment, and the typical conserved myristoylation and palmitoylation sites are enclosed by red boxes

Pairwise protein sequence comparisons among SsCBLs showed that the lowest similarity was 47.2% (between SsCBL04 and SsCBL03/06), the highest similarity was 92.5% (between SsCBL02 and SsCBL03), and the average was 61.8% (Table S2). Multiple alignments of the SsCBLs protein sequence revealed that all nine SsCBLs have three typical conserved EF-hand domains, and a conserved V-F-H-P-N motif is present at the end of the first EF-hand domain (Fig. 1). Four SsCBLs (SsCBL01, SsCBL04, SsCBL05, and SsCBL08) harbor conserved myristoylation and palmitoylation sites (M-G-C-X-X-S/T) in their N-terminal regions (Fig. 1), which have crucial roles in protein aggregation, stability, and trafficking [33, 34].

### Cis-elements analysis of *CBL* genes in *S. spontaneum*

Analysis of the *cis*-elements in the upstream promoter region of *SsCBLs* was carried out. The most frequently identified cis-elements were light-responsive, phytohormone responsive, and putative stress-responsive, as shown in Fig. S1. All *SsCBLs* except *SsCBL05* contain an abscisic acid-responsive element (ABRE) and all *SsCBLs* except *SsCBL01* contain a methyl jasmonate responsive element (JARE). Other phytohormone-responsive *cis*-elements such as ethylene-responsive element (ERE), auxin response factor (ARF), salicylic acid-responsive elements (SARE), and gibberellin responsive element (GARE) were identified in most *SsCBLs*. Putative stress-responsive *cis*-elements, such as the dehydration-responsive element (DRE), low-temperature responsive element (LTR), and MYB transcription factor binding site involved in drought-inducibility were also identified. The G-Box *cis*-element involved in light response was enriched in the promoters of five out of the nine *SsCBLs* (*SsCBLs01*, *SsCBLs02*, *SsCBLs03*, *SsCBLs06,* and *SsCBLs08*).

### Analysis of Ka/Ks values of the CBLs

To analyze the selection pressure of *SsCBL* genes in evolution, the ratios of nonsynonymous to synonymous substitution (Ka/Ks) among *SsCBLs* and their orthologous genes in *S. bicolor* were estimated. The results revealed that the Ka/Ks value of *CBL* gene pairs between *S. spontaneum* and *S. bicolor* was less than 1 (Fig. S2), which suggests that the *CBL* gene family went through strong purifying selection after the split between *S. spontaneum* and sorghum, and these genes are functionally conserved.

### Phylogenetic analysis of *CBL* genes in *S. spontaneum* and other representative angiosperms

**Fig. 2:**
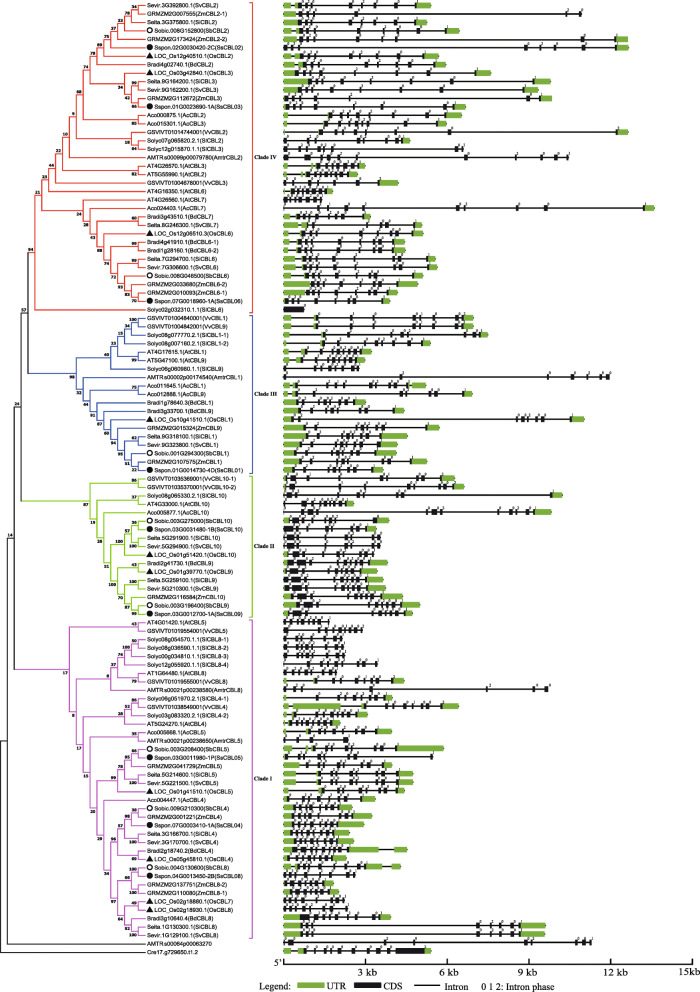
Schematic and phylogeny diagram for intron/exon organization of *CBL* genes from 13 species of plant

**Fig. 3:**
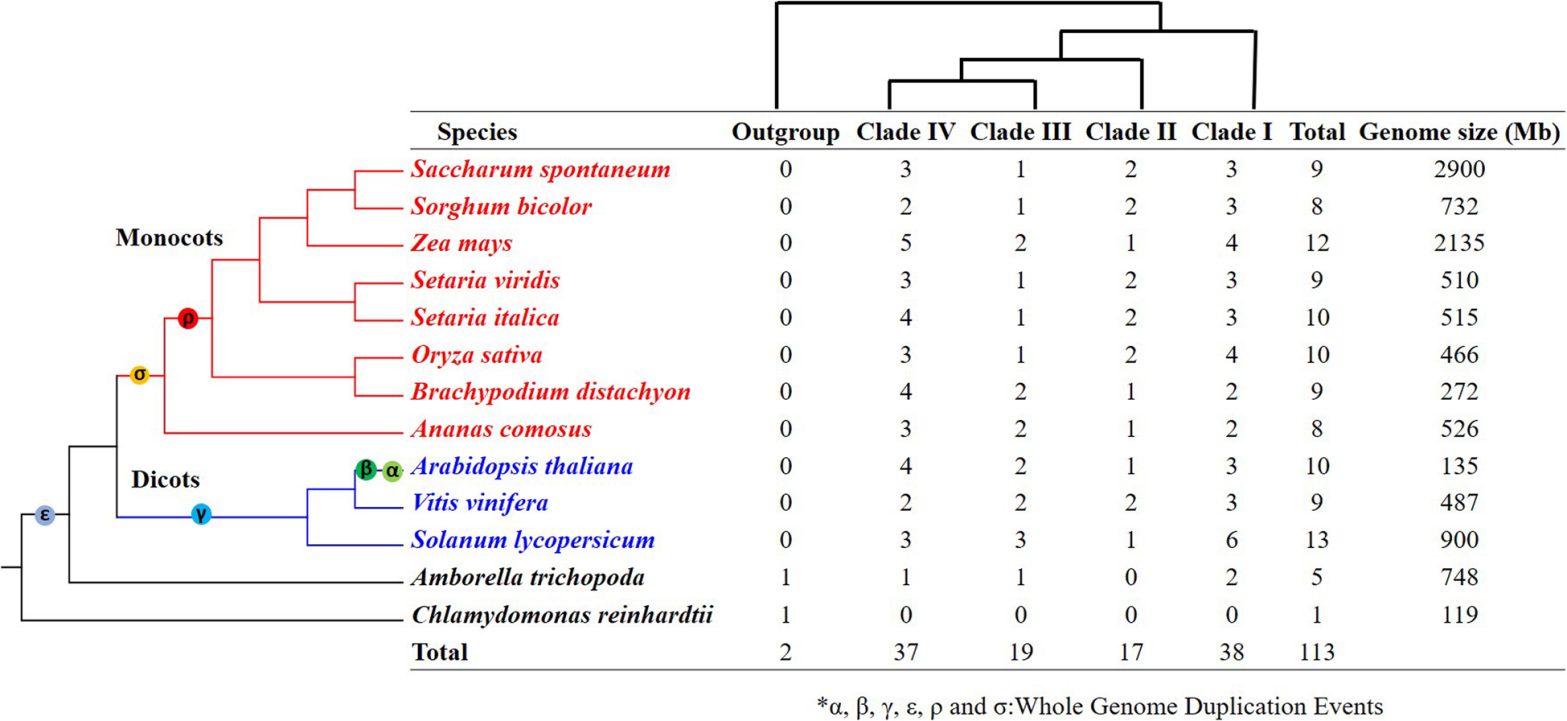
Phylogenetic relationships of *CBL* families based on the available data for angiosperms. The number of *CBL* genes and the genome size of each species are shown

To have a better understanding of the phylogenic evolution of the *CBL* gene family, a total of 112 *CBL* genes, identified from 12 representative angiosperms (8 monocotyledons, 3 dicotyledons, and *Amborella trichopoda*) were used to construct a phylogenetic tree, while selecting a *CBL* gene from *C. reinhardtii* as the outgroup (Fig. 2). The 112 *CBL* genes included 9 from *S. spontaneum*, 8 from *S. bicolor*, 12 from *Z. mays*, 9 from *S. viridis*, 10 from *S. italica*, 10 from *O. sativa*, 9 from *B. distachyon*, 8 from *A. comosus*, 10 from *A. thaliana*, 9 from *V. vinifera*, 13 from *S. lycopersicum* and 5 from *Amborella trichopoda* (Fig. 3). The results suggested that the total count of *CBL* genes in a plant species is not proportionate to the size of its genome.

The 112 *CBL* genes belonging to various species could be categorized into four clades (I, II, III, IV) (Fig. 2). *A. trichopoda*, the earliest diverging angiosperm contained 5 *CBL* genes, whereas, in dicots and monocots, the total count of *CBL* genes varied from 8 to 13, suggesting that the *CBL* gene family has undergone gene expansion, and this gene expansion is mainly attributed to the whole-genome duplications (WGDs) in both the dicot and monocot lineages (Figs. 2 and 3). Clades I, III, and IV contained *CBL* genes from all 12 angiosperms, suggesting that the ancestors of these genes predated the split of angiosperms. Moreover, the *CBLs* from *S. spontaneum* often clustered together with those from gramineous plants, especially *S. bicolor*. These results are consistent with *S. bicolor* being the closest relative of sugarcane.

### Exon/intron organization of the *CBL* family in *S. spontaneum* and other angiosperms

To analyze the intron-exon structure and evolution of the *CBL* genes in different species, cDNA sequences were mapped onto their genomic sequence. According to the results, the exon number of *CBL* family in these species varied from 1 to 12, with most *CBLs* (76 out of 113, 67.3%) containing 8 exons, indicating that the last common ancestor (LCA) of *CBLs* in angiosperms had 8 exons (Fig. 2, Fig. S3). *CBL09* and its orthologous genes in clade II had an extra exon compared with other clades, which presumably originated after an exonization event, a main driving force causing exon-intron structural differences in homologous genes [35]. The *CBL* gene size in clade IV varied the most among the four clades. The *CBL* gene structure in each subfamily was similar, and the exon sizes of the examined *CBLs* remained relatively conserved. However, the sizes of the genes varied widely, which is majorly attributed to different intron sizes or intron insertion (Fig. 2). The intron phases of the splice sites of these *CBL* genes were analyzed based on phases 1, 2, and 0 representing alternative splicing occurring after the 1st, 2nd, and 3rd nucleotide of the codon, respectively [36]. The results indicated that splicing phases of almost all introns in the same relative positions were identical, suggesting that the splicing phase of the *CBL* genes was highly conserved during evolution (Fig. 2).

### Expression analysis of *SsCBLs* in different tissues at different stages

**Fig. 4:**
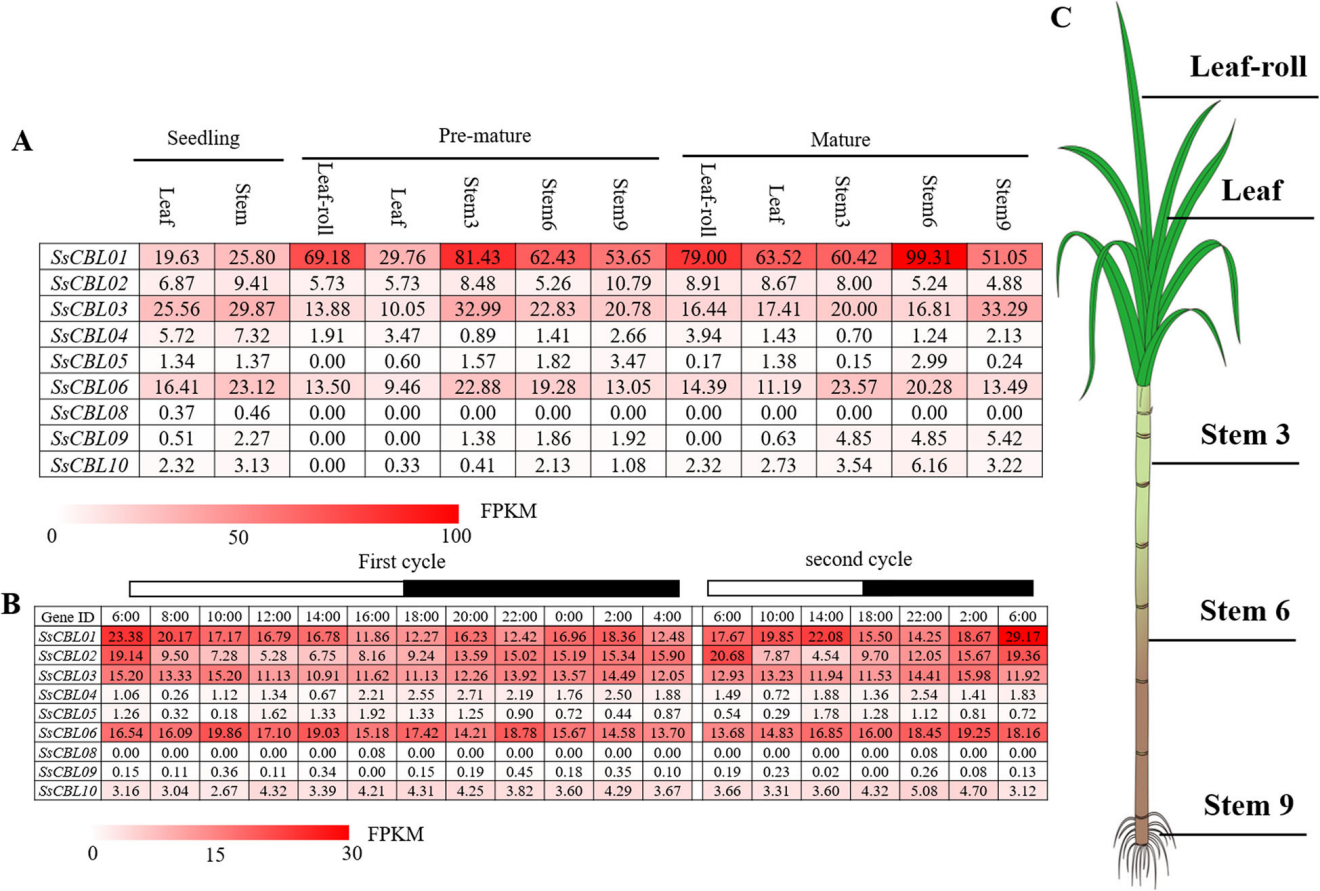
The pattern of *SsCBL* gene expression based on FPKM in *S. spontaneum*. (A) in different tissues of different stages; (B) during the diurnal cycles; (C) schematic diagram of *S. spontaneum* plant to show the tissues sampled. The FPKM is shown as the mean of three biological replicates

Transcriptome profiles of *CBLs* in *S. spontaneum* were analyzed based on RNA-seq data for investigating the expression patterns of all *SsCBL* genes in different tissues at different stages (Fig. 4A, Table S3). *SsCBL01* showed the highest expression level among all *SsCBLs*; moreover, the expression level at the pre-mature stage or mature stage was higher than that at the seedling stage. Both *SsCBL03* and *SsCBL06* showed a higher degree of expression in the stem compared to the leaf. *SsCBLs* in clade I and clade II, including *SsCBL04/05/08/09/10* all showed very low or undetectable levels of expression in the tissues examined, indicating that their role is rather limited in growth and development.

### Expression analysis of *SsCBLs* during the circadian rhythms

*CBLs* had been reported to respond to light in Arabidopsis [37]. To analyze the expression patterns of *SsCBLs* during diurnal cycles, transcriptome profiles of the mature leaves in *S. spontaneum* at 2 h intervals within 24 h and at 4 h intervals within another 24 h were investigated. The expression levels of *SsCBLs* in clade I and clade II were very low or undetectable (Fig. 4B, Table S4), further supporting their limited role and is consistent with the expression pattern at different developmental stages. It is noteworthy that the expression of *SsCBL02* was regulated by circadian rhythm, with the peak expression level in the morning and the lowest in the middle of the day (Fig. 4). This can be explained by the effects of light on phytohormone activity; the hormone cycle is directly related to the calcium signaling cascade which plays a feedback role in the hormone signaling pathway [38].

### Expression analysis of *SsCBLs* under K^+^-deficient stress

**Fig. 5:**
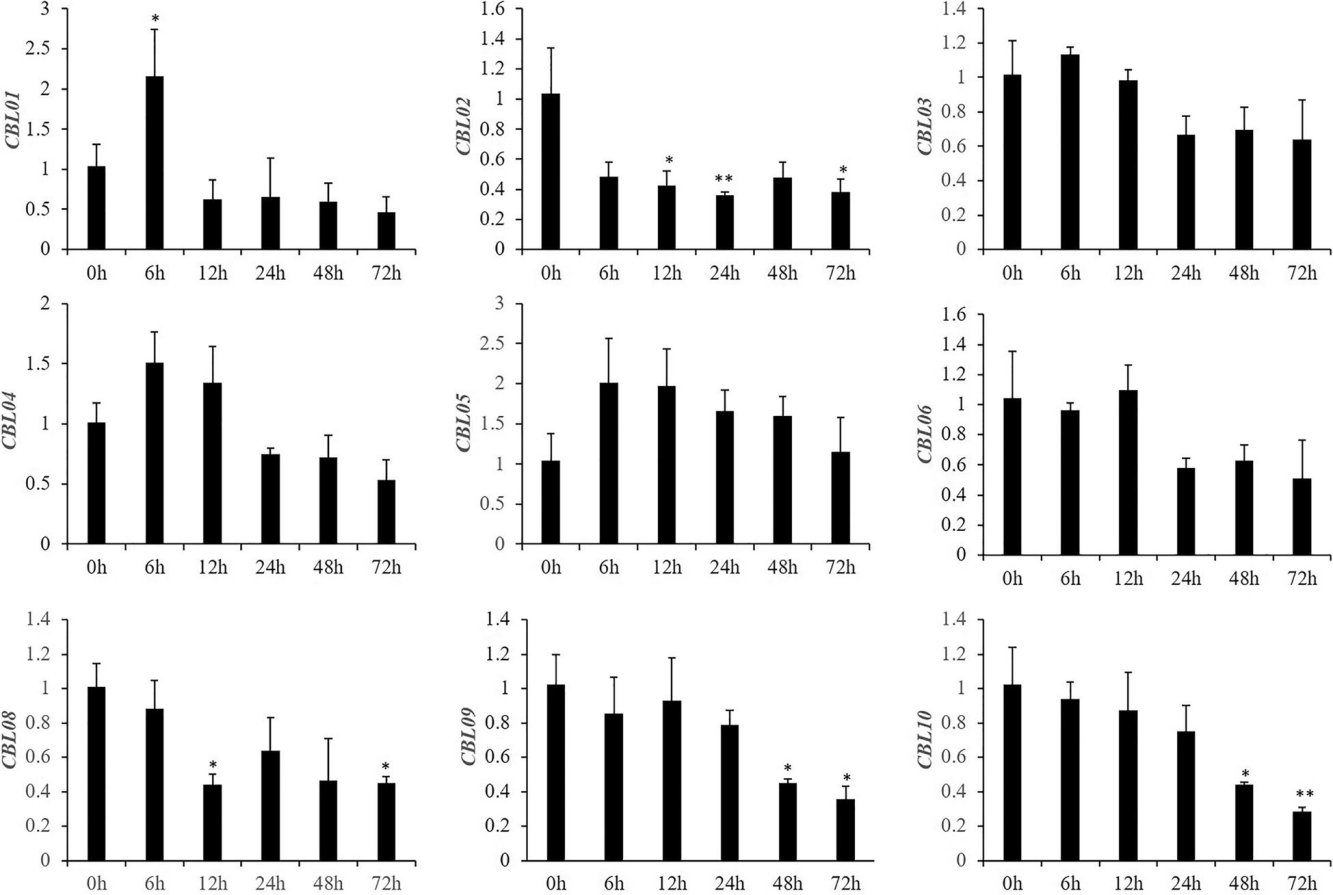
The relative expression levels of *SsCBL* genes measured by RT-qPCR under low K^+^ stress in the sugarcane hybrid YT55. The relative expression level was shown as the mean of three biological replicates and three technical replicates, * and ** respectively represent a significant difference at *p* ≤ 0.05 and *p* ≤ 0.01 based on Student’s *t*-test

*CBLs* play vital roles in decoding the signals triggered by environmental stimuli, such as K^+^ deprivation or salt stress in Arabidopsis, rice, and maize [13, 27, 39]. Accordingly, we investigated the expression patterns of the nine *SsCBLs* under K^+^ starvation by RT-qPCR. Generally, low K^+^ stress moderately altered the transcription of *SsCBLs* (Fig. 5). The nine *SsCBLs* could be categorized into two groups based on their expression patterns. One group contained *SsCBL01*/03/04/05 and showed increased expression at 6 h with decreasing expression with the prolongation of low K^+^ stress. The other group contained the *SsCBL02/06/08/09/10* genes that showed reduced expression under low K^+^ stress. The expression levels of *SsCBL01* increased significantly at 6 h but decreased at 12 h, 24 h, 48 h, and 72 h under K^+^ starvation, suggesting that *SsCBL01* may play a valuable role in the response to low K^+^ stress in *S. spontaneum*. These results also indicated that calcium signaling is an early event in the stress signaling pathway [38].

### Subcellular localization of SsCBL01

**Fig. 6:**
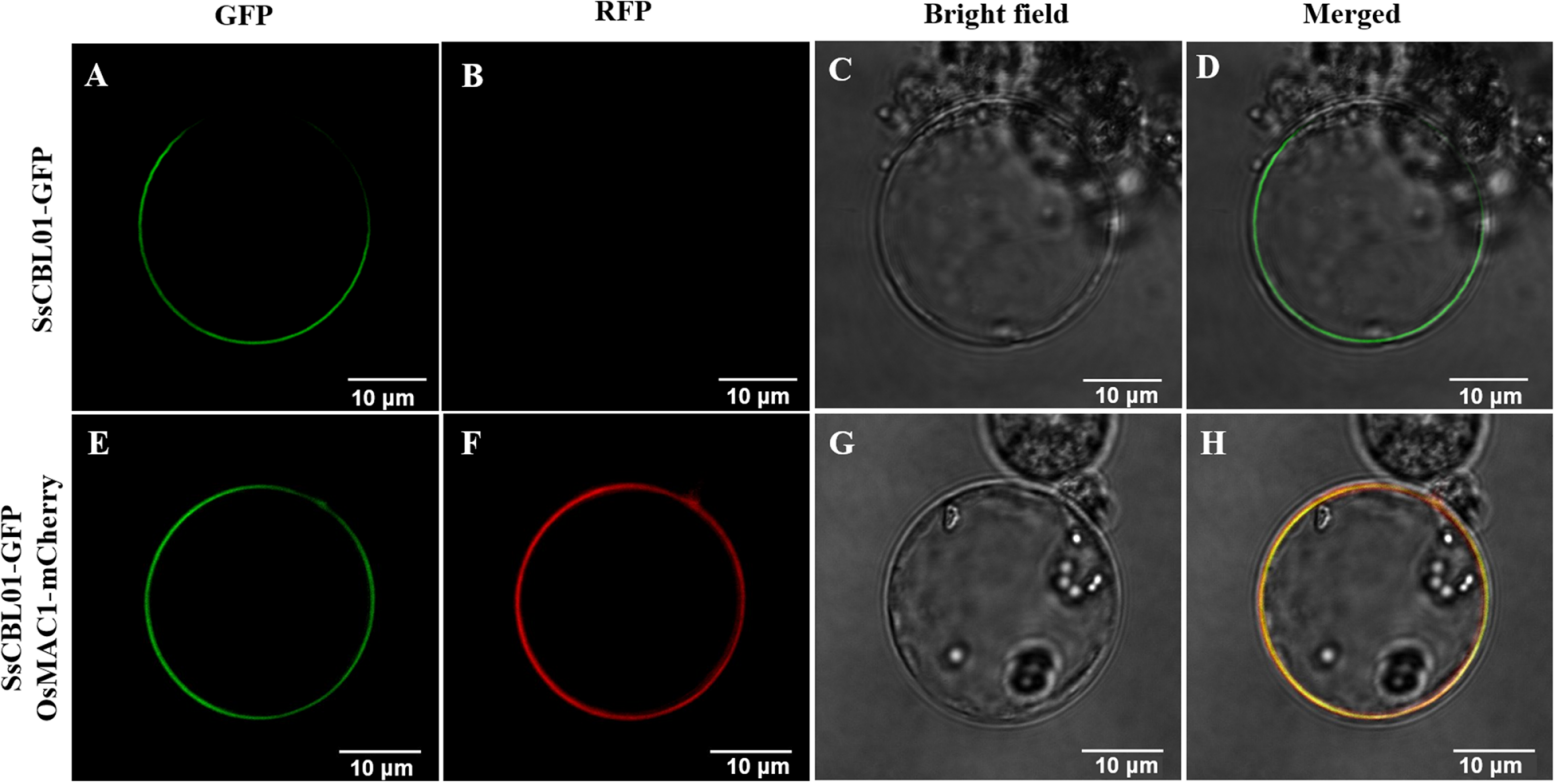
SsCBL01 localized at plasma membrane. SsCBL01 protein was fused with GFP. OsMAC1 protein was fused with mCherry. SsCBL01-GFP was individually expressed (A-D) or co-expressed with OsMAC1-mCherry (E-G) in rice protoplasts. The OsMAC1-mCherry was used as a plasma membrane localization marker. Scale bar = 10 μm

*SsCBL01* harbors a conserved myristoylation site in the N-terminal region, which is one of the characteristics of cell membrane localization. To confirm whether *SsCBL01* localizes on the cell membrane, a fusion construct of *SsCBL01* with green fluorescent protein (GFP) was used for transient transformation in the rice protoplast. The results showed that the control GFP was detected throughout the cells, while the *SsCBL01*-GFP fluorescence signal was only observed on the plasma membrane, suggesting that *SsCBL01* is a plasma membrane protein (Fig. 6). This is also consistent with the subcellular localization of the homologous *CBL1* gene in Arabidopsis and rice [18, 27].

### Functional analysis of *SsCBL01* in the defective yeast mutant

**Fig. 7:**
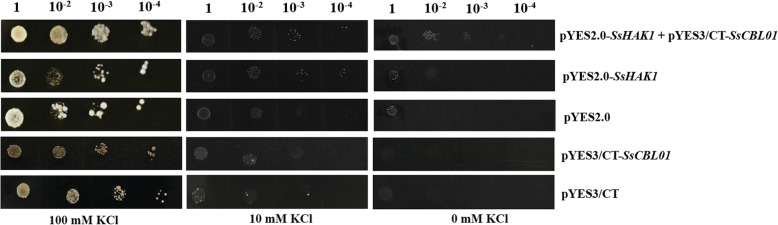
Phenotypic identification of the yeast mutant strain *R5421* transformed with *SsCBL01* and *SsHAK1* on medium with different concentrations of potassium

Expression pattern analysis showed that *SsCBL01* may have an integral role in response to stress caused by low K^+^ levels. Heterologous expression of *SsCBL01* in yeast showed that there was no obvious difference in growth between the yeast strain transformed with *SsCBL01* and the empty vector (Fig. 7), suggesting *SsCBL01* alone could not absorb K^+^. Our previous study showed that *SsHAK1* could partly recover K^+^ absorption under K^+^ starvation in the yeast mutant [40]. To study the regulatory effect of *SsCBL01* on *SsHAK1*, we constructed co-transformed yeast with both genes and observed the progress of their growth in SC/−ura medium with 100 mM, 10 mM, and 0 mM KCl. The results showed that the growth of the yeast transformed with both *SsHAK1* and *SsCBL01* was better than the yeast transformed with *SsHAK1* at 100 mM, 10 mM, and 0 mM KCl (Fig. 7). All these results suggested that *SsCBL01* alone could not absorb K^+^ but it could promote K^+^ absorption by *SsHAK1*.

## Discussion

As calcium sensors in plants, the *CBL* gene family has been found to regulate potassium stress in Arabidopsis, rice, and maize [4, 5, 8]. However, the *CBL* gene family in *S. spontaneum* has not yet been characterized. This work identified nine *CBL* genes from *S. spontaneum*; these genes together with 104 orthologous *CBL* genes from 12 plant species, and an outgroup was used to create a phylogenetic tree to study their evolution. Analysis of the expression pattern of *SsCBLs* in various tissues at individual developmental stages, during the circadian rhythms, and on exposure to low K^+^ stress was performed to study the functional divergence of *SsCBLs*. Heterologous expression in yeast revealed *SsCBL01* positively regulates K^+^ uptake by *SsHAK1*.

### Evolution of *CBL* gene family in *S. spontaneum* and representative angiosperms

WGD or polyploidy is considered to be a key trigger in the evolution of angiosperms [41, 42]. Extensive analysis of the entire Arabidopsis genome sequence revealed two recent WGDs (named α and β) in the cruciferous lineage and a triplication event (γ) that may be shared by all core dicotyledonous plants [43, 44]. Monocots have experienced two WGDs (named σ and ρ) [45], and recent research found that pineapple (*A. comosus*) had one lesser WGD event (ρ) compared with other grasses (Poaceae) [46]. Angiosperms underwent an even earlier ancient WGD event (ε) during evolution [47]. *A. trichopoda* has attracted much attention since it is the earliest angiosperm known to evolve separately from other angiosperms. The above WGD information in angiosperms along with the phylogenetic analysis of 112 *CBL* genes made it possible to study the gene evolution. The *CBL* genes from 12 angiosperms can be divided into four clusters in duplicated descending order: clade I, clade II, clade III, and clade IV.

The *CBL* genes in four clades were distributed unevenly, and *CBL* gene family members from dicotyledon and monocotyledon species were always found to be clustered in different subfamilies in all four clades. These results indicated that the *CBLs* might have undergone divergent evolution to adapt to drastic changes in the environment. The gene structure in clade I was the most conserved among the four clades, which was consistent with clade I being the most ancient clade. In clade II, all angiosperms except *A. trichopoda* contained *CBL* genes, indicating that the last common ancestor (LCA) of the *CBLs* in clade II originated after the divergence of *A. trichopoda* from the angiosperms. The gene expansion seen in clades I, II, and IV was mainly ascribed to the γ WGD in the dicot and the σ WGD in the monocot lineages; besides, the ρ WGD also led to *CBL* expansion in clades I and II in grasses (Poaceae). Clade III only contained one *CBL* gene family member, *CBL01*, and all 12 representative angiosperms, including *A. trichopoda,* had this gene, suggesting the importance and conservation of *CBL01*. In clade IV, the *CBL* gene structure varied the most among the four clades, which was also consistent with the phylogenetic analysis showing clade IV as the latest branch during evolution.

### Gene expression and functional divergence of CBLs in *Saccharrum*

Expression pattern analysis can shed light on the potential functions of the *CBL* gene family. In our study, the *CBL* genes in clade I and clade II were either not expressed or expressed to a very low degree in tissues at different developmental stage and in response to circadian rhythms, while the *CBL* genes in clade III and clade IV were the predominantly expressed genes. Clades I and II appeared earlier than clades III and IV based on the rooted phylogenetic tree. These results suggested that functional redundancy and divergence may have occurred in the *CBL* family during evolution. According to the phylogenetic analysis, *SsCBL01* was an important and conserved gene. This was further confirmed by transcriptome analysis since *SsCBL01* was the predominantly expressed gene among the nine *SsCBL* genes at different developmental stages as well as during circadian rhythms. The expression of *SsCBL02* is regulated by the circadian rhythm. In Arabidopsis, *AtCBL2* transcription was also found to be influenced by illumination and AtCBL2 responded to light signals by interacting with the SNF1-related protein kinase AtSR1 [37]. These results indicated that *SsCBL02* may be involved in signal transduction in response to light.

As a whole, K^+^ starvation had a moderate effect on the expression levels of the *SsCBL* genes. Similarly, the *CBL* genes in cotton also had similar expression patterns under K^+^ deficiency [6]. These results indicated that multiple *SsCBL* genes most likely regulate the response to low K^+^ stress in *S. spontaneum*. Strikingly, the expression of *AtCBL1* and *AtCBL9* was found to be stable under low-potassium conditions [17]. Therefore, those *SsCBLs* not induced by potassium deficiency may also be involved in the adaptation to potassium deficiency in *S. spontaneum*. The expression of *AtCBL10* in roots was observed to be moderately reduced under potassium deprivation [48]. These results suggest that the constitutive expression of some *CBL* genes may be sufficient to transmit calcium signals to downstream targets in response to low potassium stress in plants. The potassium content in roots and shoots was measured at 0 h, 6 h, 12 h, 24 h, 48 h, and 72 h under low potassium stress. It is interesting to note that the potassium content both in the shoots and roots did not change significantly under low K^+^ stress within 72 h (Fig. S4). This could be explained by reduced K^+^ concentrations in the external environment triggering a Ca^2+^ signal in the cells of the plant root, which is then recognized and relayed by CBLs to activate K^+^ channel proteins or potassium transporters, leading to enhanced uptake and transport of K^+^ in the plants over the short term [3, 16].

### Gene structure of *SsCBL* and function of *SsCBL01* in response to low K^+^ stress

All *SsCBL* genes except *SsCBL09* have eight exons, similar to *CBL* genes in other monocots and eudicots, which suggests conservation of *CBL* gene structures in different plant species. Moreover, all *SsCBL* genes contain three typical conserved EF-hand motifs, which bind to Ca^2+^ to transduce calcium signals. These properties are also strongly reminiscent of those in Arabidopsis and rice [5]. The conserved structure of *CBL* family members in different plants may imply similar modes of interaction with their target proteins, such as CIPKs.

Studies have shown that *CBLs* play key roles in response to low potassium stress in Arabidopsis and rice [16, 27]. In this study, low K^+^ stress had a moderate effect on the transcription of *SsCBLs*, with *SsCBL01* expression up-regulated and then down-regulated under K^+^ starvation. Heterologous expression of *SsCBL01* in yeast showed that *SsCBL01* alone could not mediate K^+^ uptake Further studies found that *SsCBL01* could regulate *SsHAK1* and enhance K^+^ uptake mediated by *SsHAK1* under both low potassium and normal potassium supply conditions. As in Arabidopsis, rice, and barley, CBL1 has been shown to interact with CIPK23 and phosphorylate AKT1, thereby activating its K^+^ uptake function [16, 27, 49]. Lee et al. [50] found that AtCBL1 could interact with different CIPKs, such as CIPK6, CIPK16, and CIPK23 to activate AtAKT1. The CBL1-CIPK23 complex can also phosphorylate AtHAK5 and up-regulate its activity to facilitate absorption of K^+^ in the roots of Arabidopsis [51]. In this study, it was found that SsCBL01 could enhance the K^+^ absorption-promoting activity of SsHAK1. However, whether this enhancement is due to the interaction between SsCBL01 and SsCIPKs in *S. spontaneum* needs further investigation.

## Conclusions

Sugarcane cultivars are polyploid interspecific hybrids with large and complex genomes. In this study, nine *SsCBL* genes were initially identified in *S. spontaneum* using comparative genomics The systematic evolutionary analysis revealed that *CBL* gene families have undergone gene expansion and strong purifying selection. Gene structure and protein sequence analysis showed that SsCBLs were conserved and contained three typical calcium*-*binding EF-hand domains. Expression pattern analysis in different tissues at different developmental stages and during the circadian rhythms revealed the functional divergence of *SsCBLs*. Functional verification in yeast suggested that the cell membrane-localized SsCBL01 alone could not absorb K^+^ but it positively regulated K^+^ absorption by the potassium transporter SsHAK1. In summary, this study provides a comprehensive view of the *CBL* gene family and robust candidate *SsCBLs* for the further study of functional mechanisms in *S. spontaneum*.

## Methods

### Plant materials, growth conditions and yeast mutant strain

*S. spontaneum* SES208 (2*n =* 8x = 64, from Jisen Zhang’s laboratory of Fujian Agriculture and Forestry University, FAFU) and the sugarcane commercial hybrid Yuetang 55 (YT55, bred by Institute of Bioengineering, Guangdong Academy of Sciences) were used in this study. SES208 was grown in plastic pots with soil in a greenhouse following standard growing procedures. YT55 was grown in plastic pots with an improved Hoagland nutrient solution.

The yeast mutant *R5421* (*Saccharomyces cervidinus*) is a potassium-deficient strain and is mainly used for the identification of potassium transporters, potassium channels, or sodium pumps. The strain grows normally in medium containing 100 mM K^+^, and rather slowly in medium containing 5–10 mM K^+^. However, when the concentration of K^+^ in the medium was less than 0.5 mM, R5421 cells were not able to grow.

SES208 tissues were sampled to investigate the pattern of expression at various stages of development and at different points in the diurnal cycle. To analyze the pattern of expression at different developmental stages, samples including leaf and stem from 35-day-old plants of SES208 (seedling stage), leaf roll, leaf, upper stem (i.e., Stem3), central stem (i.e., Stem6), and lower stem (i.e., Stem9) from plants at 9 (pre-mature stage) and 12 (mature stage) months of age were collected. The internodes on the *S. spontaneum* stalk were numbered from top to bottom. A previously described approach was followed for the collection of tissues [52], and three biological replicates were collected for each sample. To investigate the expression patterns in response to circadian rhythms, mature SES208 plant leaves were gathered at an interval of 2 h within the first 24 h starting from 6:00 a.m. on March 2, 2017. In the next 24 h, the collection was carried out at an interval of 4 h. The tissue collection method was the same as described previously [46], and three biological replicates were collected for each sample.

To investigate the expression pattern under conditions of low potassium stress, the hybrid sugarcane variety YT55 was bred at a normal potassium level (3.0 mM KCl) for 20 days in plastic pots with Hoagland nutrient solution (the solution was changed once a week) in greenhouse conditions (temperature: around 26 °C; relative humidity: 60–80%; light cycle: 12–12 h light-dark cycle), and subsequently transferred into the K^+^-deficient nutrient solution (0.1 mM KCl) for exposure to low K^+^ stress. Mixed root tissues from six plants in a pot (a biological replicate, a total of three biological replicates were gathered) was sampled at 0 h, 6 h, 12 h, 24 h, 48 h, and 72 h, respectively following stress treatment, then frozen immediately in liquid nitrogen and stored − 70 °C for total RNA extraction.

The potassium contents in the roots and shoots of YT55 under low potassium stress at 0 h, 6 h, 12 h, 24 h, 48 h, and 72 h were measured. The plant tissues of roots and shoots were sampled (six plants in a pot represented a biological replicate, a total of three biological replicates were gathered). These samples were placed in an oven at 105 °C, fixed for 2 min, and then dried to constant weight at 70 °C. Plant samples were crushed in a mill and then passed through a 20-mesh sieve. A total of 0.2 g sample was weighted and digested by the wet ashing method of H_2_SO_4_-H_2_O_2_ on a cooker. After digestion, the volume was adjusted to 250 mL. The potassium content (mg/g, dry weight) of each treated sugarcane plant tissue was measured using a Model 425 flame photometer (Sherwood, UK).

### Identification of CBL family genes in *S. spontaneum* and other angiosperms

The genome sequences of *S. spontaneum* were downloaded from NCBI (https://www.ncbi.nlm.nih.gov/nuccore/QVOL00000000) [32]. In the light of the earlier reports, the protein sequences of 10, 10, and 12 *CBLs* from *A. thaliana*, *Z. mays*, and *O. sativa* [1, 4, 8] were applied as queries to search putative *CBL* gene family members in the genome of *S. spontaneum* as well as 11 selected angiosperm genomes using the BLASTp program. The 11 angiosperms included 7 monocotyledons (*Z. mays, S. bicolor*, *Setaria italic, A. comosus, S. viridis*, *B. distachyon,* and *O. sativa*), 3 dicotyledons (*A. thaliana*, *S. lycopersicum,* and *V. vinifera*), and *Amborella trichopoda*, and genome sequences were downloaded from Phytozome V12.1 (https://phytozome.jgi.doe.gov/pz/portal.html). Sequences with E-values<e^− 10^ and identities higher than 70% were selected as CBL candidates. Then, the identified CBLs were queried against the Pfam (https://pfam.xfam.org) and Conserved Domain Database (CDD) (https://www.ncbi.nlm.nih.gov/Structure/bwrpsb/bwrp-sb.cgi) databases for conserved domains verification. In addition, a *C. reinhardtii CBL* was used as an outgroup.

### Sequence analysis of CBL family from *S. spontaneum*

Prediction of the properties of the CBL proteins from *S. spontaneum* and *S. bicolor* (*S. bicolor*) was carried out using ExPASy (https://web.expasy.org/compute_pi/). TBtools software was used to extract the GFF3 files of *CBL* gene family members, and the gene structures were mapped and visualized by TBtools [53]. The subcellular location of the CBL proteins was predicted by the online tool WoLF PSORT (https://www.genscript.com/wolf-psort.html). To analyze the type and distribution of cis-elements in *CBLs*, a 2 kb sequence in its upstream promoter region was selected and submitted to the online tool Plant CARE (http://bioinformatics.psb.ugent.be/-webtools/plantcare/html/) for the prediction of cis-elements in promoters.

### Phylogenetic and evolutionary analysis

The 113 CBL protein sequences from 12 angiosperms and an outgroup were aligned using ClustalW 2.0 by pairwise and multiple alignments with default parameters [54]. A phylogenetic tree of the *CBL* family was created based on the alignment results using MEGA7.0 [55]. The evolutionary history was inferred by using the Maximum Likelihood method based on the JTT matrix-based model [56]. Assessment of the reliability of the internal branches of the phylogenetic tree was completed using 1000 times bootstrap trials, displaying the percentages next to the branch.

The Ka (non-synonymous substitution rate), Ks (synonymous substitution rate), as well as the ratio of Ka to Ks of the eight pairs *CBL* orthologs from *S. spontaneum* and sorghum were estimated using Easy_KaKs calculation program (https://github.com/tangerzhang/FAFUcgb/tree/master/easy_KaKs).

### Expression profiling of CBLs in *S. spontaneum* based on RNA-seq and RT-qPCR

For investigating the pattern of expression at various stages of development and circadian rhythm, RNA from all tissues was extracted by an RNA extraction kit and employed to create cDNA libraries according to the protocol described by TruSeq™. The Illumina Hiseq 2500 platform was used to sequence the RNA-seq libraries with 100 nt paired-end. The *S. spontaneum* AP85–441 genome was used as a reference genome [32]. The raw reads were first filtered by trimming the adapter and the low-quality sequences, including reads with unknown bases of over 10% and those containing more than 50% of the nucleotides with Q-value ≤5. Then, the obtained clean reads were mapped to reference sequences. Quantitative analysis of RNA-seq was performed through Trinity (https://github.com/trinityrnaseq/trinityrnaseq/wiki), the transcriptional expression level for the individual genes was quantified by the FPKM value (fragments per kilobase of exon per million fragments mapped) using RESM in Trinity [57]; these data can be downloaded from http://sugarcane.zhangjisenlab.cn/sgd/html/index.html.

To assess the expression patterns of *CBLs* in YT55 at low K^+^ stress, 1 μg RNA extracted from the roots of YT55 under low K^+^ stress was reverse-transcribed using the PrimeScript RT reagent kit (Monad Biotech, Suzhou, China) to cDNA. Subsequently, real-time quantitative PCR (RT-qPCR) was carried out using the cDNA and SYBR Green Realtime PCR Master Mix (TOYOBO, Japan) on an ABI 7500 real-time PCR system. The specific RT-qPCR primers of the *CBLs* (Table S5) were designed by online tools Integrated DNA technologies (https://sg.idtdna.com/PrimerQuest/Home/Index), utilizing two constitutively expressed genes, β*-actin* and the *eukaryotic elongation factor 1a* (*eEF-1a*) as an internal control to normalize the gene expression levels. Three technical replicates for each sample were conducted. The relative expression levels of each *SsCBL* gene in samples treated with low K^+^ stress for different times were calculated using the 2^-△△Ct^ method [58].

### Subcellular localization

According to the CDS sequence of *SsCBL01*, primers (Table S6) containing enzyme digestion sites were designed to amplify the full-length cDNA of *SsCBL01*. cDNA synthesized by reverse transcription of YT55 RNA sample under low potassium stress for 6 h was used as the template. The PCR product was recovered and fused in frame with the coding region of green fluorescent protein (GFP) in the pBWA(V) HS-Glosgfp vector, to create the *SsCBL01*-GFP fusion construct controlled by the CaMV 35S promoter. The binding product was converted into *E. coli*-competent *DH5α* cells. Positive clones were chosen for PCR amplification and confirmed via sequencing, then the GFP fusion construct was employed for the transient transformation in rice protoplast. The transmembrane protein OsMAC1 was fused with mCherry [59]. The OsMAC1-mCherry construct was used as a plasma membrane localization marker. GFP and RFP fluorescence signals were observed using a confocal laser microscope (Nikon C2-ER) after dark culture on MS medium at 28 °C for 48 h and examined at 488 nm/640 nm (excitation) making use of an argon laser with an emission band at 510 nm/675 nm.

### Expression vector construction and heterologous expression of *SsCBL01* in yeast

Cloning of the full-length cDNA of *SsCBL01* was carried out using YT55 cDNA. The PCR product was recovered and was ligated using In-Fusion enzyme (TaKaRa Biotechnology Co., Ltd., Dalian, China) to the yeast expression vector pYES3/CT. The PCR products were converted into *E. coli*-competent *JM109* cells and monoclonal colonies were selected for PCR. The plasmid confirmed to be carrying *SsCBL01* and the empty vector pYES3/CT were transformed into the yeast mutant strain R5421 competent cells to screen positive colonies in SD/−trp medium. Positive yeasts were incubated in a liquid medium overnight to saturation before adjusting the yeast concentration to OD600 = 0.8. Yeast strains (R5421) with the empty vector pYES3/CT and *SsCBL01* were employed for gradient dilution and inoculated in SD/−trp media containing 0 mM, 10 mM, or 100 mM KCl for 3–5 days.

### Co-transformation of *SsCBL01* and *SsHAK1* in yeast mutant

In our previous study, we constructed the yeast expression vector pYES2.0-*SsHAK1* for investigating the function of *SsHAK1* [40]. To study the regulatory effect of *SsCBL01* on *SsHAK1*, pYES2.0-*SsHAK1* and pYES3/CT-*SsCBL01* were co-transformed into the yeast mutant *R5421* and inoculated in SD/−ura/−trp medium to screen positive strains. Yeast strains (*R5421*) transformed with empty vector pYES2.0, pYES2.0-*SsHAK1,* and pYES2.0-*SsHAK1* + pYES3/CT-*SsCBL01* were utilized for gradient dilution and inoculated in SC/−ura medium with 100 mM, 10 mM, or 0 mM KCl for 3–5 days.

## Supplementary Information

**Additional file 1.**

**Additional file 2.**

**Additional file 3.**

**Additional file 4.**

**Additional file 5.**

**Additional file 6.**

**Additional file 7.**

## Data Availability

All RNA-seq related data can be downloaded from the sugarcane database website (http://sugarcane.zhangjisenlab.cn/sgd/html/index.html). The *S. spontaneum* genome project [32] was deposited into Genbank with accession numbers: QVOL00000000.
