# Supplementary material for: Comparative phylogenetic analysis of CBL reveals the gene family evolution and functional divergence in Saccharum spontaneum
Source: BMC Plant Biol. 2021 Aug 23;21:395. doi: 10.1186/s12870-021-03175-3 (PMC8383383; doi:10.1186/s12870-021-03175-3)
Supplement: Supplementary file 1 — Additional file 1. [file 12870_2021_3175_MOESM1_ESM.docx]

**
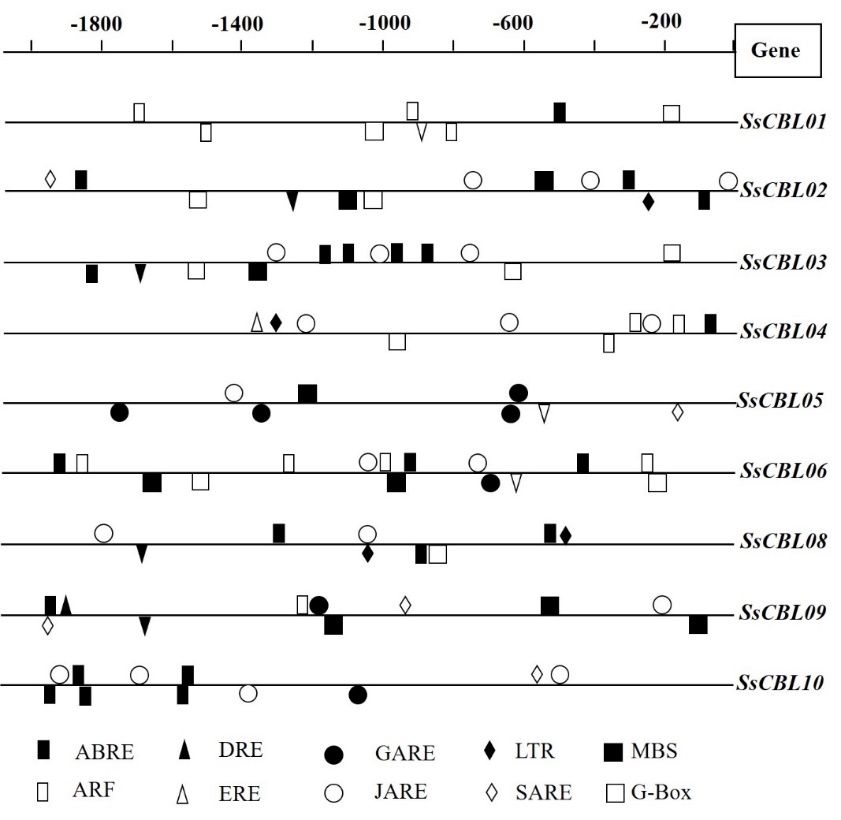
**

**Fig. S1** Phytohormone responsive, putative stress responsive and light responsive *cis*-elements in the 2 kb 5’-upstream promoter regions of *SsCBL* genes. The lines represent 5’-upstream regions of SsCBL genes. The *cis*-elements located in the reverse and forward strand were indicated below and above the lines respectively. Ten figures were used to represent different types of *cis*-elements.

**
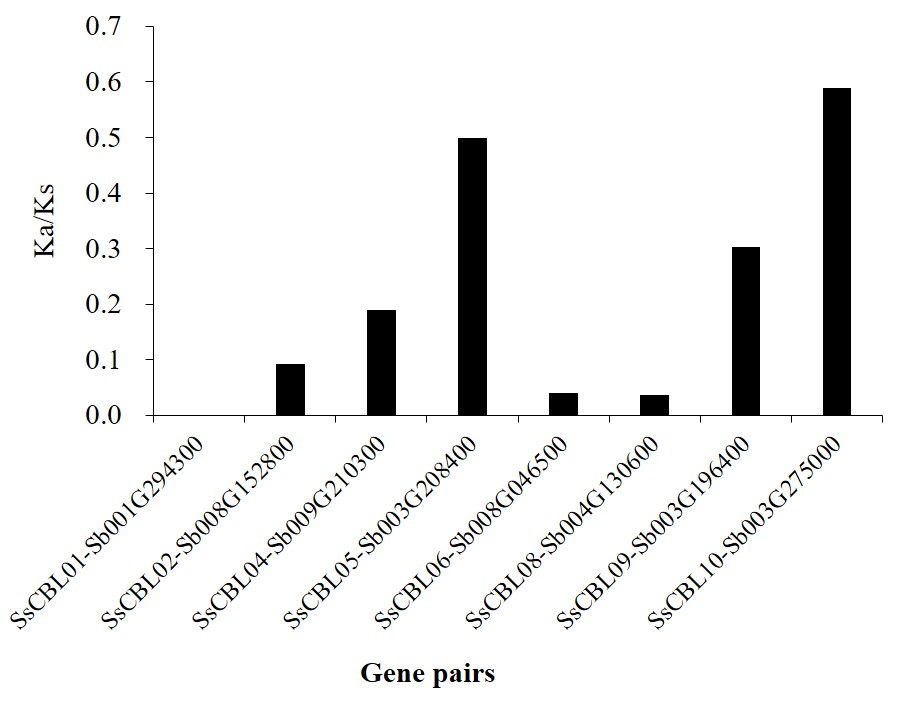
**

**Fig. S2** Nonsynonymous (Ka) and synonymous (Ks) substitution ratios of *SsCBLs* and orthologous genes in sorghum.

**Fig. S3** The proportion of different number of exons in all *CBLs* from 13 plant species


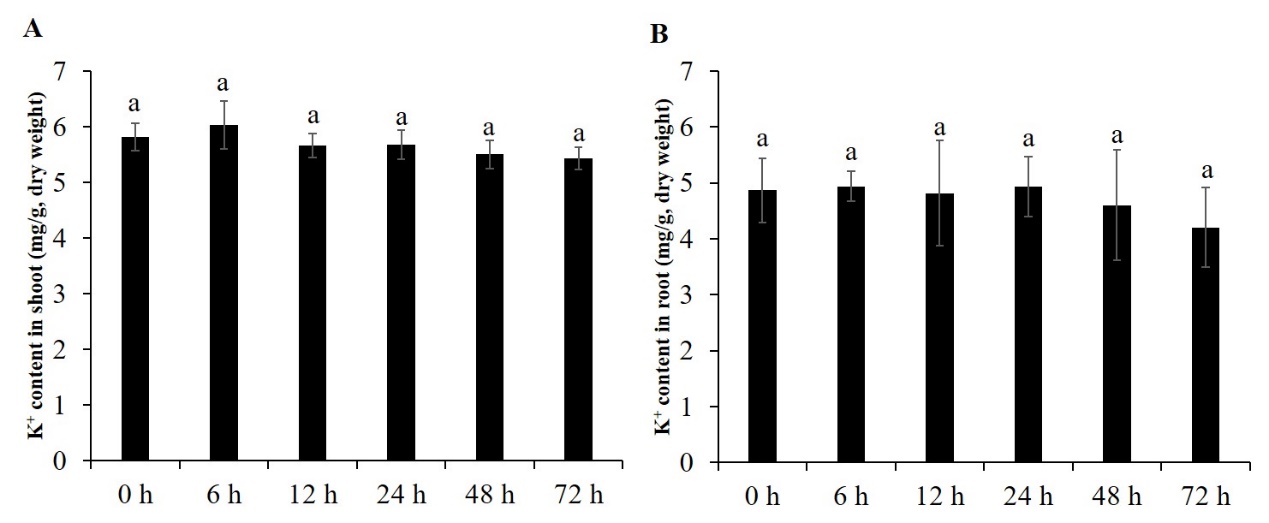


**Fig. S4** Potassium content in shoot and root at different time under low K^+^ stress. (A) K^+^ content in shoot; (B) K^+^ content in root, letter “a” on the bar chart indicates no significant difference at *p*＜0.05.
